# Treatment of the Asphyxia Caused by Chloroform

**Published:** 1860-01

**Authors:** Cyrus B. Smith


### ARTICLE XIII.

### *Treatment of the Asphyxia caused by Chloroform.*

Messrs. Editors:

I noticed in a late number of the Philadelphia *Medical and Surgical Reporter*, the statement that Langenbeck, of Berlin, successfully performed the operation of tracheotomy for asphyxia and apparent death from chloroform; hence the following:

During the past winter, I have had occasion to perform a number of experiments upon animals—cats and dogs principally. To reduce the animals to a state of get-at-ableness, and to prevent interruption to my operations, I have generally administered chloroform; but, to produce complete anæsthesia, particularly in a cat, necessitates very large doses; and I often found that, when I had arrested all the voluntary motions of the animal, the effect was made permanent by the stoppage of the involuntary motions. I observed, too, when the animal lay upon its back, while under the full influence of anæsthetics, that the respiration was often stopped, or else impeded; and when it lay upon its breast, with the mouth turned downward, this did not happen. I could find no explanation for this, except upon the supposition that the tongue fell backward from its natural position, in such a manner as to close the air-passages leading to the lungs, thus preventing the entrance of air into the organs of respiration. I have never noticed this, while the animal possessed any voluntary motion, or power sufficient to hold the tongue in its normal position during respiration. Often, too, when an animal stopped breathing, respiration was immediately resumed, if not too long stopped, by drawing the tongue forward with forceps, partly out of the mouth, and sometimes resorting to artificial respiration. Since the learning of this fact, when performing experiments upon such subjects, I have always confined the tongue partly out of the mouth, and by so doing my animals always live, unless they die from causes extraneous to the results of anæsthesia. I think, if due attention is given to these facts, that most, if not all, of the animals which die by anæsthetic cause, during experiments, may be saved; which is a great desideratum, in the neighborhood of an experimenter or a medical college, where numerous experiments render it difficult to obtain them.

Now, was tracheotomy necessary in the case in which Langenbeck operated? I think not, and its success adds to the truth of this assertion. If he had drawn the tongue forward, partly out of the mouth of his patient, I think the result would have been the same. What other explanation for the success of the operation can be given? I am not aware that pseudo-membrane, or other obstruction, is developed in the air-passages by the administration of anæsthetics. The obstruction must have been between the mouth and cricoid cartilage, and, without doubt, that obstruction was the tongue.

Generally, patients undergoing any operation, from the extraction of a tooth to the amputation of a limb, while under the influence of anæsthetics, are either placed horizontally upon the back or in a reclining position, so that if the patient lose all muscular power, the tongue would naturally fall downward and backward—thus preventing the passage of air, which requires but little change in the position of the tongue. It is not often that we hear of the death of a patient, while possessing any voluntary power. I think one reason why ether is less fatal than chloroform, is that the anæsthetic effect of ether is not often pushed to the extent to which chloroform is given, ether being less powerful in effect than chloroform.

I am surprised at the want of knowledge in the medical profession upon these simple facts; and I am satisfied that many of the deaths of persons thus asphyxiated by anæsthetics, might have been prevented by a knowledge and application of them. CYRUS B. SMITH.

[*Boston Med. and Surg. Jour.*

